# Mechanisms of Growth and Hydrogen Permeation of Zirconium Nitride Film on Zirconium Hydride

**DOI:** 10.3390/ma16010349

**Published:** 2022-12-30

**Authors:** Wenke Wang, Guoqing Yan, Zhaohui Ma, Jiandong Zhang, Lijun Wang, Zhancheng Guo

**Affiliations:** 1 National Engineering Research Center for Environment-Friendly Metallurgy in Producing Premium Non-Ferrous Metals, GRINM Group Corp., Ltd., Beijing 101407, China; 2 State Key Laboratory of Advanced Metallurgy, University of Science and Technology Beijing, Beijing 100083, China; 3 GRINM Resources and Environment Tech. Co., Ltd., Beijing 101407, China; 4 General Research Institute for Nonferrous Metal, Beijing 100088, China; 5 Beijing Engineering Research Center of Strategic Nonferrous Metals Green Manufacturing Technology, Beijing 101407, China

**Keywords:** zirconium hydride, zirconium nitride film, growth law, hydrogen permeation resistance, in situ nitridation

## Abstract

Nitride film as a hydrogen permeation barrier on zirconium hydride has seldom been studied. In this work, the zirconium nitride films were prepared on zirconium hydride in an atmosphere of N_2_ and N_2_ + H_2_ at 500~800 °C, with a holding time of 5 h and 20 h, and the mechanisms of film growth and hydrogen permeation were analyzed. The results showed that the film growth was mostly influenced by the temperature, followed by the reaction atmosphere and the holding time. The hydrogen could increase the nitrogen diffusivity during the formation of zirconium nitride films. The in situ nitriding conditions were optimized as 800 °C, N_2_ + H_2_ atmosphere, and 5~20 h. The chemical composition of ZrN-based films was mainly comprised of Zr and N, with a minor content of O. In addition, the film exhibited a major phase of ZrN, accompanied by the coexistence of ZrO_2_, ZrO, ZrN(NH_2_), and ZrN_0.36_H_0.8_, as well as O-H and N-H bonds based on the XPS analysis. The as-prepared ZrN base films in the present study exhibited superior hydrogen permeation resistance to other ZrO_2_ films previously reported. The hydrogen permeation resistance of the films could be attributed to the following mechanisms, including the chemical capture of hydrogen by the above-mentioned compounds and bonds; the physical barrier of continuous and dense film incurred from the volume effect of different compounds based on Pilling–Bedworth model and the different nitrogen diffusion coefficients at different temperatures.

## 1. Introduction

Compared with solar and chemical energy cells, nuclear reactor power supply had significant advantages in space energy, such as high power and long life. At present, the representative space nuclear reactor was TOPAZ-II; of Russia, with zirconium hydride as a core moderator [1,2]. The main advantages of zirconium hydride include high hydrogen density [3], low neutron capture cross-section, and excellent thermal conductivity [4,5,6]. Zirconium hydride was employed as the neutron moderator in the core of space nuclear reactors, with hydrogen as a functional element due to its excellent performance in decelerating fast neutrons. Hydrogen content was the key parameter when using Zirconium hydride as the carrier of hydrogen.

Hydrogen will escape from zirconium hydride due to the ZrHx ↔ Zr + x/2/H_2_ reaction equilibrium in-service temperature and atmosphere [7,8]. Thus, developing hydrogen permeation barrier films on the surface of zirconium hydride was an effective approach to prevent hydrogen from escaping and ensure the effectiveness of the moderator. Numerous research has been conducted on the preparation of hydrogen permeation barrier on stainless steel, such as oxide films of Y_2_O_3_, Cr_2_O_3_, and Al_2_O_3_ [9,10,11,12,13,14], carbide films of SiC [15], nitride films of AlN, Si_3_N_4_, BN, TiAlN [16,17,18,19,20], and multi-element composite films [21,22] by such methods as magnetron sputtering, chemical vapor deposition, arc deposition, and pack cementation. Compared with stainless steel, zirconium hydride moderator in nuclear reactors generally showed a honeycomb structure, making it difficult to achieve uniform coating by physical vapor deposition; besides, film preparation at high temperature such as embedding infiltration were not applicable for zirconium hydride due to the hydrogen loss.

Abundant studies have been reported with regard to zirconium hydride, including zirconium oxide films by in situ oxidation, complex oxide films by sol-gel, oxide films by micro-arc oxidation, and chromium/chromium oxide films by electroplating [23,24,25,26,27,28,29,30,31,32,33,34,35]. The ZrO_2_ film containing N was prepared by in situ reaction of the decomposition atmosphere of urea with zirconium hydride [32,33]. However, films with ZrN as the major phase of zirconium hydride have been seldom studied and the mechanisms of growth and hydrogen permeation resistance of ZrN film are not clearly revealed. At present, zirconium oxide film made by in situ reaction performs the best in application, however, the main problem of the zirconium oxide film was the inevitable occurrence of cracks and spalling due to the phase transformation from t-ZrO_2_ to m-ZrO_2_ at high temperatures. Based on the excellent hydrogen permeation resistance of nitride, the preparation and performance of zirconium nitride films on zirconium hydride was studied in this paper.

The current methods of nitriding mainly include gas nitriding and plasma nitriding by N_2_, N_2_ + H_2_, and NH_3_ [36,37,38,39,40,41,42,43,44]. Among them, gas nitriding could generate nitride films on the surface of metal or alloy by reacting with nitrogen with the gas sources of pure nitrogen or decomposed ammonia at high temperature and was relatively simple and easy to operate. Plasma nitriding with a faster nitriding speed required electrodes and a high-voltage electric field. However, since the zirconium hydride moderator exhibited a complex structure, the films were hard to form on the electric contacting point of the sample surface due to the difficult electrode design and manufacture.

The objective of this study was to obtain a dense and continuous film with good hydrogen permeation resistance and durability on the surface of zirconium hydride. In this work, the zirconium nitride films were prepared on zirconium hydride under the conditions of temperature from 500 °C to 800 °C, holding time of 5 h and 20 h, and atmosphere of N_2_ and N_2_ + H_2_. The possibility of preparing zirconium nitride-based film by in situ reaction method with N_2_ + H_2_ gas was investigated, and the influence factors of film growth and film properties were studied.

## 2. Materials and Methods

### 2.1. Preparation of Hydrogen Permeation Barrier

Zirconium hydride (H/Zr = 1.8) was prepared by the research group in the National Engineering Research Center for Environment-friendly Metallurgy in Producing Premium Non-ferrous Metals. Prior to the usage, zirconium hydride was polished to 6.5 μm with sandpaper step by step, the sandpaper grit was made of aluminum oxide, and then ultrasonically rinsed in 99.7% ethanol for 300 s.

Firstly, the influence of temperatures (500 °C, 600 °C, 700 °C, and 800 °C) on the film growth of zirconium hydride was investigated with a holding time of 20 h in pure N_2_ atmosphere, and hydrogen loss of the matrix was discovered. After shortening the reaction time and adding H_2_ to N_2_, the hydrogen loss of the matrix was significantly reduced. The film was prepared by sealing the zirconium hydride sample and gas in a quartz tube. Three zirconium hydride samples of Φ20 × 3 mm were put into the quartz tube of 1.27 × 10^−4^ m^3^ filled with about 1.02 × 10^−3^ mol of N_2_ under 20 kPa pressure, and the amount of N_2_ for growing films of 5 μm thickness consumed about 4.2 × 10^−4^ mol of N_2_, much less than the nitrogen provided, followed by heating to the specified temperatures at a rate of 1 °C/min and keeping for a period of time. The film was prepared when the samples were cooled to room temperature. Table 1 lists the specific experimental scheme for the preparation of the hydrogen permeation barrier on zirconium hydride.

### 2.2. Film Characterization

A scanning electron microscope (SEM, Hitachi S-4800, Tokyo, Japan) was employed to observe the surface and cross-section morphologies of the film; the sample surface was sprayed with gold to ensure electrical contact. X-ray diffraction (XRD, Smartlab KD2590N, Tokyo, Japan) was employed to investigate the phase of the surface layer. The composition and elemental distribution of the film were analyzed by Auger electron spectroscopy (AES, ULVAC PHI-700, Chigasaki, Japan). The hydrogen content of the zirconium hydride before and after in situ reaction was determined by 836 Series Elemental Analyzer (LECO, St. Joseph, MO, USA). The measurements in Kadri [45,46] and Rasheed’s [47,48] study, such as XRD analysis and linear fitting of data, were referenced in this study.

### 2.3. Diffusion Coefficient Calculation

The diffusion law of N and O during the film growth was explored based on the distribution of elemental content with a depth by AES and Fick’s second law. Fick’s second law is shown in Equation (1) [49]:

(1) $$\frac{\partial c}{\partial t}=k\frac{\partial^{2}c}{\partial x^{2}}$$

where *c* represents the concentration at various places during diffusion; *t* is the time, s; *x* is the diffusion distance, cm; *k* is the diffusion coefficient, cm^2^/s.

Based on the film deposition and relevant literature [50,51], the diffusion coefficient was calculated based on the formula as follows:

(2) $$k=\frac{1}{t}(\frac{x}{{2\mathrm{erf}}^{-1}\left(\frac{c_{1}{-c}_{x}}{c_{1}}\right)}{)}^{2}$$

where *c_x_* represents the concentration at various places during diffusion; *c*_1_ is the concentration of the film surface layer.

### 2.4. Evaluation of Hydrogen Permeation Performance

The hydrogen permeation performance of nitride film was evaluated using non-destructive online gas chromatography, and the model developed by Bai S [34] and Qi S [35] were employed for data analysis. The zirconium hydride with nitride film was tested in gas chromatography equipment for 7 days at 600 °C in the atmosphere of He + 50% CO_2_. The contents of H_2_, CO, and CO_2_ in the atmosphere were analyzed every 24 h to calculate the hydrogen loss. The hydrogen content of samples before and after the chromatography experiments were determined by 836 Series Elemental Analyzer, and the results of gas chromatography were verified.

Two nitride film samples No. 9 and No. 4 in Table 1 were applied for the hydrogen permeation resistance test, marked by “nitride film 1” and “nitride film 2”. The sample of in situ oxide film previously prepared in the CO_2_ atmosphere [29] was used for comparison. The samples and their manufacturing conditions are listed in Table 2.

## 3. Results and Discussion

### 3.1. Hydrogen Loss of Zirconium Hydride Matrix after Film Preparation

**Figure 1:**
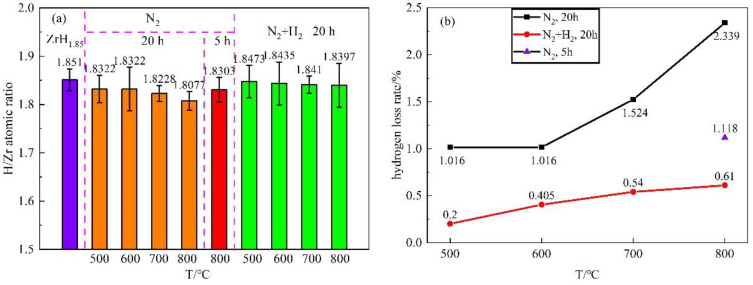
H/Zr atomic ratio and hydrogen loss rate of zirconium hydride matrix when film prepared in N_2_ and N_2_ + H_2_ atmosphere (**a**) H/Zr atomic ratio; (**b**) hydrogen loss rate.

The hydrogen contents of samples after the preparation of nitride films were evaluated by the 836 Series Elemental Analyzer. As shown in Table 3 and Figure 1, when the films were prepared in pure N_2_ atmosphere at 500 °C, 600 °C, 700 °C, and 800 °C for 20 h, the atomic ratio of H/Zr of zirconium hydride matrix decreased from the initial 1.85 to 1.8322, 1.8322, 1.8228, and 1.8077, corresponding to hydrogen loss rates of 1.016%, 1.016%, 1.524%, and 2.339%, respectively. With increasing the temperature during the film preparation, the hydrogen loss rate of the zirconium hydride matrix increased gradually, especially at 800 °C.

In order to reduce the hydrogen loss of the zirconium hydride matrix during the film preparation, the holding time was shortened from 20 h to 5 h in a pure N_2_ atmosphere at 800 °C. It can be seen that the hydrogen loss rate was reduced from 2.339% to 1.118%. In addition, hydrogen was added into nitrogen for the further reduction of hydrogen loss. Under the condition of N_2_ + H_2_ atmosphere with a holding time of 20 h, the atomic ratios of H/Zr of zirconium hydride with nitride films prepared at 500 °C, 600 °C, 700 °C, and 800 °C were 1.8473, 1.8435, 1.8410, and 1.8397, with the corresponding hydrogen loss rate of 0.200%, 0.405%, 0.540%, and 0.610% respectively. Thus, substituting N_2_ + H_2_ atmosphere for pure N_2_ can significantly reduce the hydrogen loss, probably because the added H_2_ balanced the hydrogen partial pressure of the system and inhibited the escape of hydrogen from the zirconium hydride matrix.

### 3.2. Morphologies and Chemical Composition of Nitride Film

#### 3.2.1. Film Thicknesses and Morphologies

**Figure 2:**
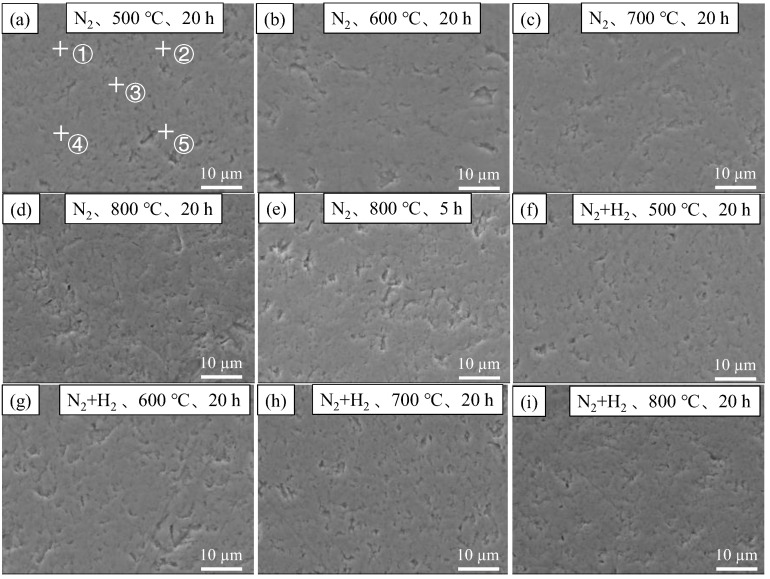
Surface SEM images of zirconium hydride with nitride film (**a**) N_2_, 20 h, 500 °C; (**b**) N_2_, 20 h, 600 °C; (**c**) N_2_, 20 h, 700 °C; (**d**) N_2_, 20 h, 800 °C; (**e**) N_2_, 5 h, 800 °C; (**f**) N_2_ + H_2_, 20 h, 500 °C; (**g**) N_2_ + H_2_, 20 h, 600 °C; (**h**) N_2_ + H_2_, 20 h, 700 °C; (**i**) N_2_ + H_2_, 20 h, 800 °C.

**Figure 3:**
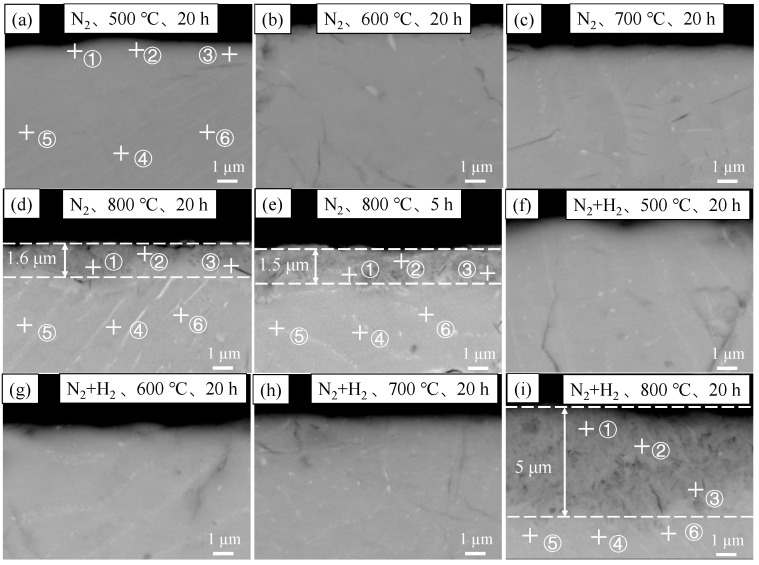
Cross-sectional SEM images of zirconium hydride with nitride film (**a**) N_2_, 20 h, 500 °C; (**b**) N_2_, 20 h, 600 °C; (**c**) N_2_, 20 h, 700 °C; (**d**) N_2_, 20 h, 800 °C; (**e**) N_2_, 5 h, 800 °C; (**f**) N_2_ + H_2_, 20 h, 500 °C; (**g**) N_2_ + H_2_, 20 h, 600 °C; (**h**) N_2_ + H_2_, 20 h, 700 °C; (**i**) N_2_ + H_2_, 20 h, 800 °C.

Figure 2 and Figure 3 show the SEM surface and cross-section morphologies of nitride film samples. Almost no significant difference was found among the surface morphologies of nitride films prepared in different conditions, indicating that the similar surface morphology before and after the film preparation. In addition, continuous and dense films without obvious defects were observed from the cross-section morphology. The film’s growth was not obvious at 500 °C, 600 °C, and 700 °C with a holding time of 20 h in both N_2_ and N_2_ + H_2_ atmospheres. However, the film growth could be visible at 800 °C, with film thicknesses of 1.5 μm and 1.6 μm when it was prepared with holding time of 5 h and 20 h in the N_2_ atmosphere and 5 μm under the condition of a holding time of 20 h in N_2_ + H_2_ atmosphere. The results indicated that temperature was the essential influencing factor, followed by the reaction atmosphere, and the time exerted the least effect on film growth, indicating that hydrogen could promote the diffusion rate of nitrogen during the film preparation.

#### 3.2.2. Elemental Composition and Distribution of the Nitride Films

**Figure 4:**
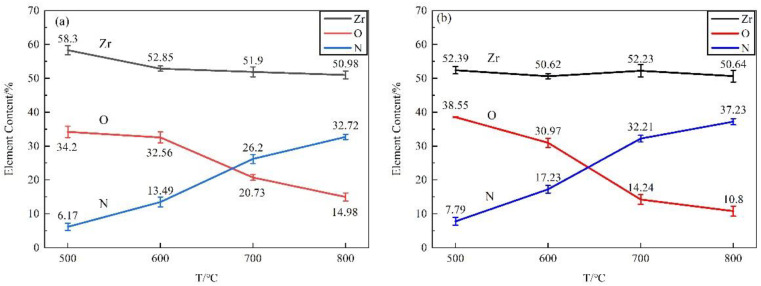
EDS analysis results of surface of zirconium hydride with nitride film (**a**) N_2_ atmosphere; (**b**) N_2_ + H_2_ atmosphere.

**Figure 5:**
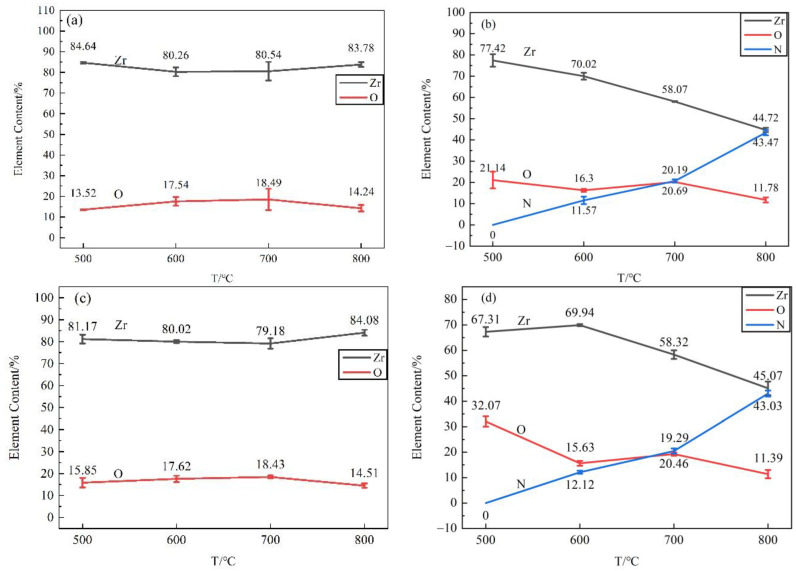
EDS analysis of cross-section of zirconium hydride with nitride film (**a**) matrix area in N_2_ atmosphere; (**b**) film area in N_2_ atmosphere; (**c**) matrix area in N_2_ + H_2_ atmosphere; (**d**) film area in N_2_ + H_2_ atmosphere.

The EDS energy spectrum of the nitride films was detected with 5 points for each surface in Figure 2, 3 points in the film area and 3 points in the matrix area for each cross-section in Figure 3. The average contents and standard errors against the temperatures were plotted in Figure 4 for the surfaces and Figure 5 for the cross-sections.

As shown in Figure 4, the major elements of the film surface were Zr, N, and O. The nitrogen content in the nitride film prepared at 500 °C, 600 °C, 700 °C and 800 °C for 20 h were 7.79%, 17.23%, 32.21% and 37.23% in the N_2_ + H_2_ atmosphere, while 6.17%, 13.49%, 26.20% and 32.72% in pure N_2_ atmosphere respectively. With increasing the temperature, the nitrogen content increased gradually, while the contents of zirconium and oxygen decreased on the surface. Compared with pure N_2_ atmosphere, the surface exhibited an increased nitrogen content in N_2_ + H_2_ mixed atmosphere, and the tendency became more obvious under a higher temperature.

As shown in Figure 5, the films prepared in both N_2_ and N_2_ + H_2_ atmospheres at 500 °C were composed of Zr and O without the detection of N, mainly due to the thin film. The detected oxygen may be attributed to residual oxygen in the atmosphere. At 600 °C and above, nitrogen was detected in the films, and its content increased with the increase in temperature. At 800 °C, the main elements of the film were Zr and N, followed by element O, indicating the film was composed of a major component of zirconium nitride and a small amount of zirconia. The elemental composition and their changing trends of the films in cross-sections with the temperatures were consistent with that of the surface, indicating little change of elemental contents in the matrix.

**Figure 6:**
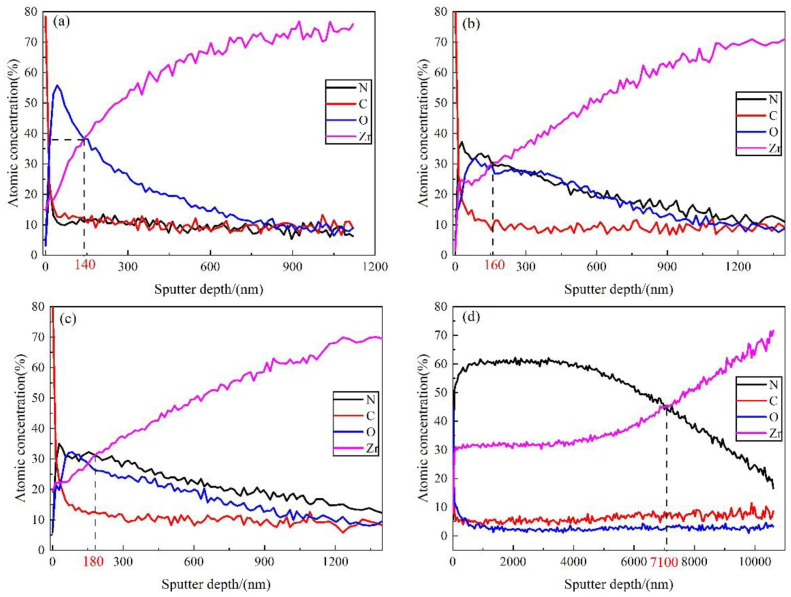
AES element distribution with sputtering depth in the film prepared in N_2_ + H_2_ at 500~800 °C for 20 h (**a**) 500 °C; (**b**) 600 °C; (**c**) 700 °C; (**d**) 800 °C.

AES method was applied to analyze the composition distribution of nitride film formed in N_2_ + H_2_ atmosphere at 500 °C, 600 °C, 700 °C and 800 °C for 20 h, and the results are shown in Figure 6. It could be seen that the major elements in the film were Zr and O at 500 °C, Zr, N, O at 600 °C and 700 °C, and Zr, N at 800 °C. These results indicated that the residual oxygen in the atmosphere would form an extremely thin zirconia film on zirconium hydride at 500 °C, showing a certain hydrogen permeation resistance effect. Zirconium hydride could react with nitrogen at 600 °C and 700 °C to generate zirconium nitride film. The film growth was significant, with a fast N diffusion rate at 800 °C, which was consistent with the SEM and EDS results. The intersection of Zr content line and N (or O) content line was used to determine the film thickness [52]. It can be seen that the film thickness was 140 nm, 160 nm, 180 nm, and 7100 nm at 500 °C, 600 °C, 700 °C, and 800 °C, respectively. Therefore, no obvious film was observed by SEM due to the thin film formed at 500~700 °C, ellipsometer method may provide a solution for its measurement precisely.

The Zr content in the film formed at 800 °C increased with the increase of the sputtering depth, while the N content increased firstly and then decreased with the highest value of about 60% at a depth of 1000 nm. The content of O decreased to almost zero when the sputtering depth was greater than 1500 nm. Thus, it can be concluded that the nitrogen exhibited a deeper diffusion depth than the oxygen, probably because the high temperature was conducive to nitrogen diffusion during the in situ reaction in the nitrogen atmosphere. The film thickness was around 2160 nm measured by AES for the film prepared in pure N_2_ atmosphere at 800 °C [53], while the film thickness reached 7100 nm at 800 °C in N_2_ + H_2_ atmosphere, indicating that hydrogen in the atmosphere exerted a significant effect on the enhancement of nitrogen diffusion rate.

#### 3.2.3. Phase Compositions of the Nitride Films

**Figure 7:**
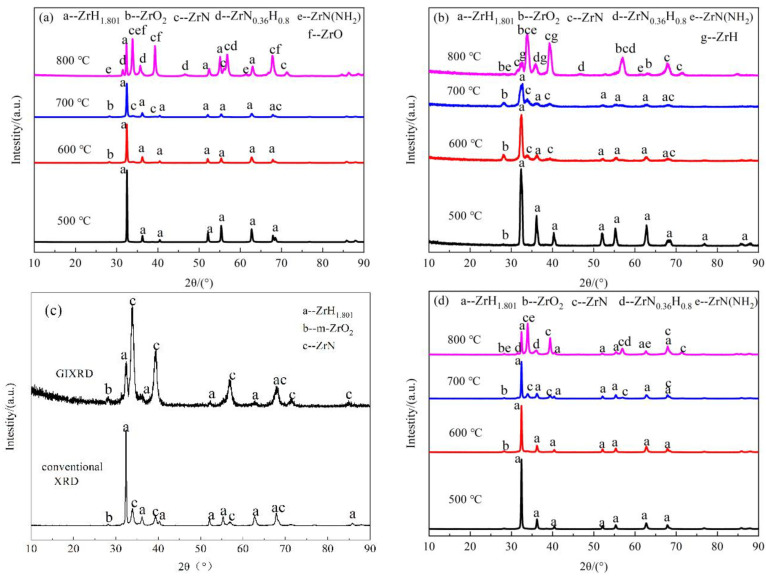

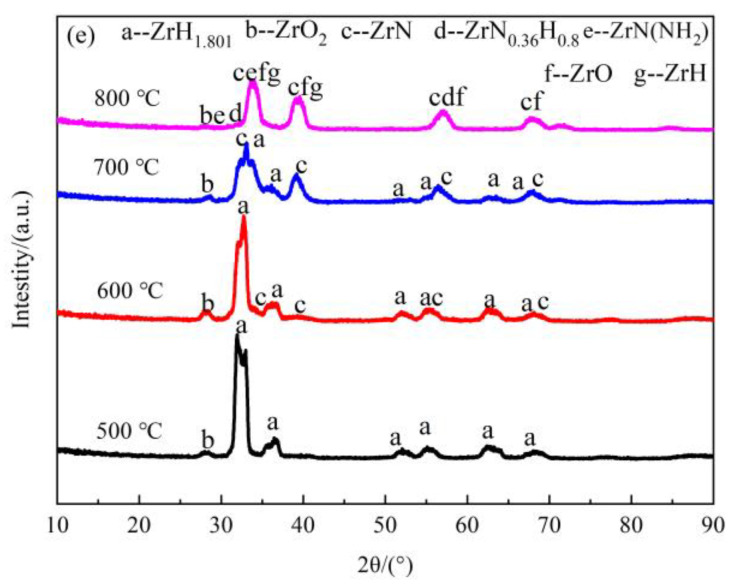
XRD spectra of zirconium hydride with nitride film prepared in different conditions (**a**) N_2_, 500~800 °C, 20 h, conventional XRD; (**b**) N_2_, 500~800 °C, 20 h, GIXRD; (**c**) N_2_, 800℃, 5 h; (**d**) N_2_ + H_2_, 500~800 °C, 20 h, conventional XRD; (**e**) N_2_ + H_2_, 500~800 °C, 20 h, GIXRD.

Figure 7 shows the XRD analysis results of nitride films on zirconium hydride prepared in different conditions. Figure 7a,b shows the XRD analysis results of nitride films prepared in pure N_2_ at 500~800 °C for 20 h; Figure 7c displays the XRD analysis results of nitride film prepared in pure N_2_ at 800 °C for 5 h; Figure 7d,e show the XRD analysis results of nitride film prepared in N_2_ + H_2_ at 500~800 °C for 20 h. The results of the phase analysis are summarized in Table 4.

The phase of conventional XRD was ZrH_1.801_ in pure N_2_ atmosphere, which was still the matrix phase. However, the m-ZrO_2_ phase was detected by grazing incidence XRD (GIXRD) at 500 °C, indicating that the oxide film was too thin to be detected by conventional XRD due to the low temperature. The temperature of the oxidation reaction was lower than that of the nitridation reaction with zirconium hydride. Compared with the characteristic peak at 500 °C, m-ZrO_2_ and ZrN phases appeared in the patterns of conventional XRD and GIXRD at 600 °C. The surface phase characteristics at 700 °C were similar to those at 600 °C, except that ZrN was detected by conventional XRD, indicating an enhanced surface nitridation reaction at a higher temperature. At 800 °C, the phases of conventional XRD of the film included ZrH_1.801_, ZrN, ZrO, ZrN(NH_2_), and ZrN_0.36_H_0.8_; ZrH and ZrO_2_ were detected by GIXRD without ZrH_1.801_ and ZrO, and the main peak changes to ZrN both by conventional XRD and GIXRD. These results indicated that the ZrN-based film was generated on zirconium hydride, and the matrix did not undergo the dehydrogenation phase transition. That is, the film with ZrN as the major phase exhibited excellent hydrogen permeation resistance.

The characteristic XRD peaks of nitride film prepared in N_2_ at 800 °C for 5 h were the same as for 20 h, but no ZrN(NH_2_), ZrN_0.36_H_0.8_, ZrH phases were observed, probably due to the short holding time, and little Zr-N-H ternary compound generated. The phase structures of nitride films prepared in an N_2_ + H_2_ atmosphere at 500~800 °C were consistent with that in a pure N_2_ atmosphere. The ZrO_2_ phase form at 500 °C in both atmospheres showed a hydrogen permeation resistance effect; The ZrN, ZrN(NH_2_), and ZrN_0.36_H_0.8_ phases formed at high temperature could reinforce the film.

XPS analysis revealed that there were Zr-O, Zr-N, O-H, N-H bonds in the film prepared in pure N_2_ at 800 °C for 20 h [53]. The mechanisms of hydrogen permeation resistance of nitride films could be revealed by combining these results with XRD results.

### 3.3. Growth Kinetics of Nitride Film

The diffusion coefficients k of N and O in the nitride film grown at 500 °C, 600 °C, 700 °C, and 800 °C were calculated using Equation (2), in which the diffusion coefficient depends on the content values of the two-points at the distribution lines in Figure 6. The parameters *x*_1_, *x*_2_, *c*_1_, *c_x_* were measured from the AES distribution lines and listed in Table 5. The diffusion front was the diffusion distance when the element content tends to be constant, as *x*_2_.

It showed that the diffusion coefficients of N and O increase gradually with the increase in temperature. At 500 °C, the diffusion coefficient of O was greater than N, and meanwhile, the diffusion of O in zirconium hydride mainly formed a thin oxide film on its surface. With the increase in temperature, the diffusion coefficient of N was greater than that of O at 600 °C and above, and nitride film was formed on zirconium hydride; at a temperature of 800 °C, the diffusion coefficient of N was much greater than that of O.

**Figure 8:**
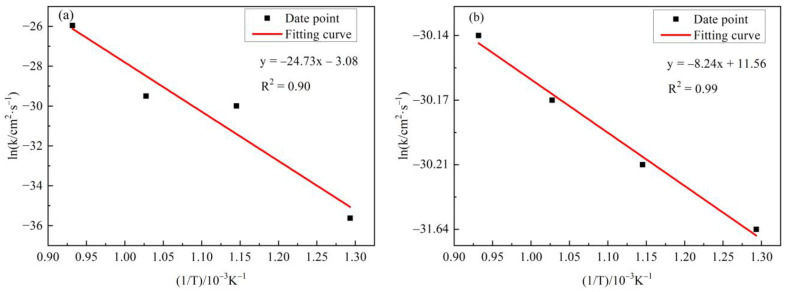
The relationship between lnk of N and O and 1/T (**a**) N element; (**b**) O Element.

The relationship between lnk and the 1/T for N and O diffusion was established based on the data in Table 5, and the results were plotted in Figure 8. A good linear relationship was shown with a correlation coefficient of 0.90 and 0.98, respectively. According to the Arrhenius equation lnk = −Q/(RT) + lnk_0_, the diffusion activation energies of N and O were 205.6052 KJ/mol and 68.5074 KJ/mol, respectively.

It is worth noting that in this study, N_2_ + H_2_ was used as the atmosphere for film preparing, and the oxygen in the film mainly came from the residue contents of H_2_O, CO_2_, O_2_ in the atmosphere and crucible, that was, the film was grown with extremely limited oxygen content. If the film growth atmosphere was changed, the diffusion laws of N and O might vary, which would be investigated in subsequent studies.

### 3.4. Hydrogen Permeation Performance and Mechanism

#### 3.4.1. Hydrogen Permeation Performance

The hydrogen permeability of the three samples in Table 2 was tested at 600 °C in 50% CO_2_ + He atmosphere for 7 days by gas chromatography and hydrogen determinator. Table 6 lists the comparison results of hydrogen loss rate for different films. It can be seen that the two nitride films exhibited slightly different hydrogen loss rates, which were much lower than that of the oxide film, indicating the better hydrogen permeation resistance properties of nitride films.

#### 3.4.2. Hydrogen Permeation Mechanisms of Nitride Film

The high hydrogen permeation resistance was attributed to its composition and structure of ZrN-based film on zirconium hydride.

Firstly, the ZrN-based hydrogen permeation barrier prepared at high temperature was mainly composed of Zr, N, and O. The major phase of the film was ZrN, and the secondary phases were ZrO_2_, ZrO, ZrN (NH_2_) and ZrN_0.36_H_0.8_. Besides, O-H bond and N-H bond were detected in the film [53,54,55]. Thus, the hydrogen permeation resistance mechanism of the film was partial because these compounds and bonds could synergistically capture the diffusing H.

Secondly, the continuity and compactness of the film also exerted a key role in hydrogen permeation resistance. Pi-ling and Bedworth proposed that whether the film formed on the metal surface had a protective effect mainly depended on the integrity of the film. The necessary condition for the integrity of the film was that the volume (*V_OX_*) of the film generated during the in situ reaction was larger than the volume (*V_M_*) of the metal matrix [56]. The ratio was denoted as PBR (Pilling Bedworth Ratio) value:

(3) $$PBR=\frac{V_{OX}}{V_{M}}=\frac{m_{OX\;}\rho_{M}}{m_{M}\;\rho_{OX}}=\frac{M_{OX\;}\rho_{M}}{M_{M}\rho_{OX}}>1$$

where $m_{OX\;}$ is the quality of the film; $\rho_{OX}$ is the density of the film; $M_{OX\;}$ is the relative molecular weight of the film;$\;m_{M\;}$ is the mass of the metal matrix; $\rho_{M}$ is the density of the metal matrix; $M_{M\;}$ is the relative molecular weight of the metal matrix.

The ZrO_2_, ZrN, ZrH_1.801_, ZrN(NH_2_), and ZrN_0.36_H_0.8_ had densities of 5.82 g·cm^3^, 7.04 g·cm^3^, 5.61 g·cm^3^, 3.70 g·cm^3^ and 6.28 g·cm^3^, and the calculated *PBR* values were 1.27, 0.90, 1.74 and 0.93, respectively. The content of ZrO_2_, ZrN, ZrH_1.801_, ZrN(NH_2_), and ZrN_0.36_H_0.8_ was 14.1%, 30.3%, 24.2%, 22.2%, and 9.1%, respectively, based on the XRD semi-quantitative analysis, with the *PBR* value of ZrN based composite film of 1.13.

The *PBR* value of ZrO_2_ was greater than 1, meeting the necessary condition of being protective. However, when the zirconia film was subject to compressive stress during a long period service, it was easy to crack and peel off. Thus, the pure zirconia film was not an ideal hydrogen permeation barrier on zirconium hydride. Although the PBR value of ZrN was less than 1, the coexistent small amount of ZrO_2_, ZrN(NH_2_), and ZrN_0.36_H_0.8_ in the film could provide adaptive compensation for ZrN volume shortage, making the *PBR* value of ZrN based composite film change to 1.13. Moreover, the film could keep continuous and compact without peeling off, which greatly improved the hydrogen permeation resistance of the film. In addition, when the application environment of zirconium hydride film was 600 °C and He + CO_2_ atmosphere, the film could self-repair adaptively to ensure the dense and continuous state of the film to make sure its protective property.

Finally, the nitrogen diffusion coefficient at 600 °C was about 57 times lower than that at 800 °C. Thus, the film preparation at a higher temperature and the usage at a lower temperature could ensure the performance of the nitride films in hydrogen permeation resistance.

## 4. Conclusions

In this work, the zirconium nitride film was prepared on zirconium hydride under conditions with temperatures from 500 °C to 800 °C, a holding time of 5 h and 20 h, and an atmosphere of N_2_ and N_2_ + H_2_. The morphologies, compositions, phase structures, growth kinetics, hydrogen permeation resistance performance were investigated, and the mechanisms of ZrN-base film were revealed.

(1) The growth of nitride films on zirconium hydride was mainly determined by temperature, followed by reaction atmosphere, and holding time. The film preparation was optimized at 800 °C in N_2_ + H_2_ atmosphere.
(2) The hydrogen content of the zirconium hydride matrix was reduced during the film preparation, and the hydrogen loss increased with the increase of temperature. In addition, the hydrogen loss was obviously reduced after shortening the preparation time and adding hydrogen to nitrogen. When the film preparation time was shortened from 20 h to 5 h in pure N_2_ atmosphere at 800 °C, the hydrogen loss rate was reduced from 2.339% to 1.118%; substituting N_2_ + H_2_ atmosphere for pure N_2_, the hydrogen loss rate was reduced from 2.339% to 0.610%.
(3) The ZrN-based films prepared at 800 °C was mainly composed of Zr, N, and O; the major phase of the film was ZrN, and the secondary phases was ZrO_2_, ZrO, ZrN (NH_2_), and ZrN_0.36_H_0.8_. The diffusion activation energies of N and O for film growth were 205.6052 KJ/mol and 68.5074 KJ/mol, respectively.
(4) Three mechanisms of hydrogen permeation resistance of nitride film were revealed, including the capture of the diffusing hydrogen, the continuous and dense structure of ZrN-based film, and the film preparation at a higher temperature than the usage.

## Figures and Tables

**Table 1:** The experimental scheme for the preparation of hydrogen permeation barrier on zirconium hydride.

No.	Atmosphere	t/h	T/°C	Test Content
1	N_2_	20	500	SEM, XRD, H content
2			600	SEM, XRD, H content
3			700	SEM, XRD, H content
4			800	SEM, XRD, H content
5		5	800	SEM, XRD, H content
6	N_2_ + H_2_	20	500	SEM, XRD, H content, AES
7			600	SEM, XRD, H content, AES
8			700	SEM, XRD, H content, AES
9			800	SEM, XRD, H content, AES

**Table 2:** Preparation conditions of three different films on zirconium hydride.

Films	Atmosphere	T/°C	t/h
nitride film 1	N_2_ + H_2_	800	20
nitride film 2	N_2_	800	20
oxide film	CO_2_	600	20

**Table 3:** H/Zr atomic ratio and hydrogen loss rate of zirconium hydride matrix when film prepared in N_2_ and N_2_ + H_2_ atmosphere.

Atmosphere	T/°C	t/h	H/Zr Atomic Ratio	Standard Deviation σ	Hydrogen Loss Rate/%
zirconium hydride			1.8510	0.0338	-
N_2_	500	20	1.8322	0.0283	1.016
	600		1.8322	0.0449	1.016
	700		1.8228	0.0164	1.524
	800		1.8077	0.0192	2.339
	800	5	1.8303	0.0256	1.118
N_2_ + H_2_	500	20	1.8473	0.0337	0.200
	600		1.8435	0.0444	0.405
	700		1.8410	0.0175	0.540
	800		1.8397	0.0456	0.610

**Table 4:** The phase of zirconium hydride with nitride film prepared in different conditions.

Atmosphere	t/h	T/°C	XRD	Phase				
N_2_	20	500	XRD	ZrH_1.801_	-	-	-	-
			GIXRD	ZrH_1.801_	m-ZrO_2_	-	-	-
		600	XRD	ZrH_1.801_	m-ZrO_2_	-	-	-
			GIXRD	ZrH_1.801_	m-ZrO_2_	ZrN	-	-
		700	XRD	ZrH_1.801_	m-ZrO_2_	ZrN	-	-
			GIXRD	ZrH_1.801_	m-ZrO_2_	ZrN	-	-
		800	XRD	ZrH_1.801_	ZrO	ZrN	ZrN(NH_2_)	ZrN_0.36_H_0.8_
			GIXRD	ZrH	m-ZrO_2_	ZrN	ZrN(NH_2_)	ZrN_0.36_H_0.8_
	5	800	XRD	ZrH_1.801_	m-ZrO_2_	ZrN	-	-
			GIXRD	ZrH_1.801_	m-ZrO_2_	ZrN	-	-
N_2_ + H_2_	20	500	XRD	ZrH_1.801_	-	-	-	-
			GIXRD	ZrH_1.801_	m-ZrO_2_	-	-	-
		600	XRD	ZrH_1.801_	m-ZrO_2_	-	-	-
			GIXRD	ZrH_1.801_	m-ZrO_2_	ZrN	-	-
		700	XRD	ZrH_1.801_	m-ZrO_2_	ZrN	-	-
			GIXRD	ZrH_1.801_	m-ZrO_2_	ZrN	-	-
		800	XRD	ZrH_1.801_	m-ZrO_2_	ZrN	ZrN(NH_2_)	ZrN_0.36_H_0.8_
			GIXRD	ZrH	ZrO/m-ZrO_2_	ZrN	ZrN(NH_2_)	ZrN_0.36_H_0.8_

**Table 5:** Diffusion coefficients of N and O in zirconium hydride at different temperatures.

Elements	T/°C	*x*_1_/nm	*x*_2_/nm	*c*_1_/at%	*c_x_*/at%	*k*/(cm^2^/s)
N	500	14	70	23.5314	9.9047	3.36 × 10^−16^
	600	28	1428	37.2274	8.5421	9.42 × 10^−14^
	700	28	1484	35.0039	11.4784	1.54 × 10^−13^
	800	912	10,608	61.7605	16.6006	5.34 × 10^−12^
O	500	42	896	55.8545	5.2977	1.82 × 10^−14^
	600	84	1540	32.0525	5.5136	7.55 × 10^−14^
	700	84	1568	32.1972	4.9837	7.89 × 10^−14^
	800	48	1680	12.0662	1.5902	8.14 × 10^−14^

Note: The diffusion time t in this study was 72,000 s.

**Table 6:** Comparison of hydrogen loss rate after testing.

Film	Hydrogen Loss Rate of Hydrogen Determinator Method/%	Hydrogen Loss Rate of Gas Chromatography Method/%
Nitride film 1	0.497	0.576
Nitride film 2	1.796	1.204
Oxide film	3.248	2.319

## Data Availability

The data presented in this study are available upon request from the corresponding author.
